# BIODEMOGRAPHY OF AGING: INSIGHTS FROM TWINS AND THE OLDEST-OLD IN DENMARK

**DOI:** 10.1093/geroni/igad104.0196

**Published:** 2023-12-21

**Authors:** Kaare Christensen

**Affiliations:** University of Southern Denmark, Odense, Syddanmark, Denmark

## Abstract

The Danish Twin Registry holds health information from surveys and registers on more than 180,000 twins born in Denmark since 1870. Life course studies of the oldest twin cohorts show moderate heritability of lifespan but a substantial genetic influence on physical and cognitive functioning throughout life, i.e., twins are growing old but not growing apart, in terms of health phenotypes. Twins experience substantial growth restriction in the last intrauterine trimester. The generalizability of aging studies of twins hinges on whether this early life exposure has long-lasting health consequences. Generally, after the infant period, twin-singleton health differences are small, and during the 20th century, they have vanished. In the older twin cohorts, sex differences in the association between number of offspring and oral health late in life provided support for the proverb known in many countries: “A tooth per child”. Twin studies of biomarkers of aging revealed that perceived age estimated by lay assessors based on photos is a strong biomarker of aging. The 1895, 1905, 1910 and 1915 Danish Birth Cohort Studies of the oldest old were established to study secular trends in the health and functioning of the very old. Among the main findings of the studies are: Exceptional longevity does not lead to exceptional levels of disability; summed over cohorts, more people are living to the highest ages and are functioning better – and this progress is seen across the spectrum of health and functioning – offering hope for continued progress in health and survival among the oldest in society.

